# State of the art: What have the pain sciences brought to physiotherapy?

**DOI:** 10.4102/sajp.v76i1.1390

**Published:** 2020-02-24

**Authors:** Romy Parker, Victoria J. Madden

**Affiliations:** 1 Pain Management Unit, Department of Anaesthesia and Perioperative Medicine, Neuroscience Institute, University of Cape Town, Cape Town, South Africa; 2 Groote Schuur Hospital, Cape Town, South Africa

**Keywords:** pain sciences, physiotherapy, clinical reasoning, assessment, treatment, education

## Abstract

**Background:**

Pain is the most common reason for patients to seek help from a health care professional. In the past few decades, research has yielded gains in the Pain Sciences - multiple fields of scientific research that, when integrated, help to clarify what causes and influences human pain.

**Objectives:**

In this article, we discuss the key areas in which the Pain Sciences have shifted the physiotherapy profession.

**Method:**

A narrative review of the Pain Sciences literature was conducted. The review analyses how the Pain Sciences have influenced physiotherapy in several categories: assessment; clinical reasoning; treatment; research rigor and building the profile of the profession.

**Results:**

Scientific research on pain has largely converged in support of three ‘game-changing’ concepts that have shifted the physiotherapy profession’s understanding and treatment of pain: (1) pain is not a signal originating from bodily tissues, (2) pain is not an accurate measure of tissue damage and (3) the plasticity of the nervous system means the nervous system itself is a viable target of treatment. These three concepts have influenced physiotherapy assessment and treatment approaches, and research design to consider pain mechanisms using patient-centred models.

**Conclusion:**

The Pain Sciences have shifted physiotherapists’ assessment and treatment approaches and shifted the status of the physiotherapy profession. Ultimately the Pain Sciences have embedded interdisciplinary teams and expanded physiotherapy practice.

**Clinical implications:**

We believe that the pain sciences should be embedded in undergraduate and postgraduate education and training of physiotherapists (including the three key concepts regarding pain) to benefit physiotherapists and their patients.

## Background

Pain is the most common reason for people to present for healthcare. The vast majority of patients treated by physiotherapists report pain, yet it is only in the past few decades that the scientific study of pain has yielded substantial progress in its treatment. The shift in focus – from studying tissue damage to studying pain itself – was arguably prompted by the recognition that the pain people report frequently does not match up with the condition of bodily tissue. Therefore, the assumption that pain reflects tissue state had to be reconsidered (Wall & McMahon 1986). Thus emerged the pain sciences – multiple fields of scientific research that, when integrated, help to clarify what causes and influences human pain. This focus on the mechanisms underlying pain has required knowledge from diverse fields – including neurology, immunology, endocrinology, epigenetics, psychology and physiology – among others. It has catalysed a seismic shift in physiotherapy and changed the underpinnings of clinical reasoning, expanding and deepening clinical practice and student training, empowering patients, and has thereby influenced research practice. This ultimately has raised the profile of the physiotherapy profession by contributing to its shifting from its origins as a profession that was reliant on expert opinion to a profession that now relies on scientific evidence.

## Key concepts that have shifted our understanding of pain

Scientific research on pain has largely converged in support of three ‘game-changing’ concepts that have fundamentally shifted physiotherapists’ understanding and treatment of pain (Box 1). The first game-changing concept is that pain is not a signal that originates from bodily tissues (Wall & McMahon 1986). Rather, pain is now thought to be a highly motivating perceptual experience that seems to be generated on the basis of a perceived need to protect bodily tissue from harm (Moseley 2007). The second game-changing concept is that pain is not a dependable indicator of tissue state. In contrast, somatosensory signalling is only one of the many, varied factors that influence the likelihood and features of pain (Moseley & Butler 2017). Furthermore, afferent signalling from peripheral tissues is subject to modulation at multiple points along its route to the brain, such that even the somatosensory signalling that arrives at the brain is a product of the nervous system’s plasticity (Woolf 2011). The third game-changing concept arises from the second: the plasticity that is inherent to bodily systems supports a reversal of sensitisation processes and represents a viable target of treatment. This is particularly important for physiotherapy, precisely because physiotherapists are experts in using movement and learning to alter and train neural pathways, thus harnessing plasticity for clinical benefit.

BOX 1 Three key concepts that have shifted physiotherapists’ understanding of pain.

1. Pain is not a signal originating from bodily tissues. Rather, it is a compelling perceptual experience generated on the basis of an apparent need to protect tissues from harm.
2. Pain is not an accurate measure of tissue damage.
3. The plasticity of the nervous system means that the nervous system itself is a viable target of treatment.

Understanding that pain seems to be a protective drive requires physiotherapists to identify the potential contributors to that protective drive. This demands a methodical mechanism-based clinical reasoning process.

At the risk of personifying the brain, we ask, ‘What makes the brain think that this part of the body needs protection?’ Because the brain is constantly processing an extraordinary amount of information from many sources, making sense of a patient’s pain requires careful consideration of a wide range of potential contributors. Clinical reasoning based on recognising collections of signs and symptoms – that is, pattern recognition – no longer suffices in this situation, because it carries a high risk of error and favours the discarding of information that violates the particular pattern (Jones, Jensen & Edwards 2008). A more complex, methodical process of reasoning is required, one that retains flexibility and accuracy, with the physiotherapist using reflection-in-action to avoid ‘missing’ subtle features of the patient’s presentation that may be contributing to pain and could be targeted with treatment. Many physiotherapists encountered this methodical, reflective, mechanistic clinical reasoning process during their early training (albeit within a biomechanical framework), but it is the scientific discovery of the many processes that can contribute to pain that has supported mechanistic clinical reasoning to become the mainstay of comprehensive, evidence-based clinical care for pain (Nijs, Van Houdenhove & Oostendorp 2010).

## The pain sciences have shifted physiotherapists’ assessment approaches

Knowledge of the science of pain has equipped physiotherapists to be able to identify a wider range of potential contributors to a patient’s pain. The list of contributors to pain translates into a longer list of possible targets for treatment. It is assumed that modifying an accurately identified contributor will also modify pain. For this process to be helpful, physiotherapists must critically engage with the full range of mechanisms that are capable of contributing to pain – which requires a truly biopsychosocial model for practice (Wijma et al. 2016). Science has moved on from the idea of psychosocial ‘yellow flags’, as a set of vulnerability factors separate from the body, to an understanding that psychological and social factors all have physiological links (Denk, McMahon & Tracey 2014). Understanding that every thought, every feeling and every interaction with the environment is linked to a chemical reaction in the neuroimmune system, with possible structural and functional consequences, increases the clinician’s insight and truly embeds biopsychosocial thinking into practice. Also, the ongoing psycho-neuro-immunological research is now expected to shed light on how psychological distress, poverty or low levels of education affect physiology (Borsook et al. 2018; Kemeny & Schedlowski 2007). As the pain sciences progress, clinicians will be better equipped to understand and identify the contributors to a person’s pain and treatments that could modify those contributors or their influence.

Physiotherapists have the opportunity to be psychologically informed and embrace the complexities of the human being and the multiple variables that affect physiology, functioning and participation. The benefits of being a *Pain-Science*-informed physiotherapist who is trained in mechanistic clinical reasoning are particularly apparent in South Africa where graduates are expected to practice in highly variable settings. A versatile yet methodical mechanistic reasoning process provides physiotherapists with a structured process to identify viable treatment options.

## The pain sciences have shifted physiotherapists’ treatment approaches

Knowledge of the science of pain also fosters patient-centred care. It supports physiotherapists and patients to collaboratively understand the contributors to their pain and, thereby, to make informed choices about treatments that could target those contributors. This selection of treatments is perhaps one of the most difficult tasks that a patient must perform when seeking healthcare, particularly if they lack understanding of biology, psychology or physiology. Patients are typically better equipped to participate in treatment selection when they understand the proposed mechanistic contributors to their pain (Lorig et al. 2001). The physiotherapist’s task is to explain their bio-psychosocially informed hypothesis about the possible contributors to the pain experienced (Nijs et al. 2010). The patient can then participate in treatment selection by weighing the advantages and disadvantages of each treatment option against their expert knowledge of themselves and their own life, as well as their own opinion on the relative importance of each proposed contributor. This empowerment of patients is fundamental to patient-centred care (World Health Organization 2008). Advances in the science of pain, which have occurred in parallel with advances in psychology, epigenetics and neuroimmunology, among others, have refocussed the attention of physiotherapists on understanding and equipping the person with pain. As we understand the complex interplay of variables that affect a person suffering from pain, we can focus our attention on the person rather than on the tissues. An additional benefit of research on pain has been to shift treatment from being single-mindedly focussed on eliminating pain to valuing the broader goal of restoring people to the life roles that are meaningful to them (Feliu-Soler et al. 2018).

The adoption of complex, mechanism-based clinical reasoning by physiotherapists has also shifted the level at which research questions are asked in the pursuit of evidence-based practice (Benton & Benton 2019; Wiles et al. 2012). Researchers and clinicians no longer engage with evidence merely to determine whether a treatment does or does not work for a certain condition. In contrast, our deeper scientific knowledge allows critical and deep engagement in *how* treatments act to potentially influence clinical outcomes. Research based on mechanism-based clinical reasoning approaches that incorporate knowledge gained through the pain sciences allows us to select treatments that are supported by clear rationale, as well as by the traditional evidence of clinical benefit. Evidence of low risk is also provided by systematic reviews and meta-analyses.

## The pain sciences have shifted the status of the physiotherapy profession

An enormous benefit for the physiotherapy profession is that it has – rather serendipitously – been thrust into the limelight by scientific research on pain. Education and exercise have been identified as two of the most effective low-risk, high-benefit treatment options for treating pain and hastening recovery. A growing body of evidence suggests that an important component of treatment for pain is explaining the contributors to pain to a patient (Foster et al. 2018). This has variably been called ‘Explaining Pain’, ‘Pain Neuroscience Education’ and ‘Therapeutic Neuroscience Education’, but the common thread is that understanding their pain helps patients to move forward towards self-management and recovery of life roles.

Importantly, communication principles emphasise providing the information the patient wants, rather than the information the clinician thinks they ought to learn. Effective education about pain has been shown to immediately improve movement (Moseley 2004) in people with low back pain, to normalise spinal inhibition of nociception in people with fibromyalgia (Van Oosterwijck et al. 2013) and to be associated with successful recovery in people with back pain (Foster et al. 2018). Uptake of this treatment approach has required a dramatic shift in the type of education given by physiotherapists, *away* from explanations focussed on structural pathology and ergonomic advice (both of which are linked to poor outcomes), and towards explanations focussed on the biopsychosocial mechanistic contributors to pain, the benefits of which are supported by scientific evidence.

A deeper understanding of pain mechanisms has reinvigorated physiotherapists’ use of exercise as a multimodal treatment strategy. Exercise is a medicine for pain (Law & Sluka 2017). Pain research has clarified that graded exercise can be used to address many different types of contributors to pain. Exercise addresses motor contributors such as deconditioning and loss of motor variability, and brings the neural signalling benefit of exercise-induced hypoalgesia (Lima, Abner & Sluka 2017; Sluka et al. 2013). Exercise improves sleep deficits that are known to be associated with pain (Nijs et al. 2018).

When used in a goal-directed, graded strategy, exercise improves mood, self-efficacy and cognition (Eime et al. 2013). Exercise that is done in a social context can mitigate social contributors to pain (Eime et al. 2013). In fact, a growing body of knowledge also suggests that exercise may even be preventative of persistent pain – perhaps via a serotonin mechanism or by regulating neuroinflammation (Grace et al. 2016; Lima et al. 2017). Importantly, an understanding of pain mechanisms changes the way exercise is prescribed: we now encourage patients to exercise in a way that is contingent not on pain but on tissue healing time.

Physiotherapists are ideally positioned to deliver both education and exercise as evidence-based, first-line treatments for pain: we have the necessary expertise, we have first-line practitioner status (in South Africa), we typically have more time with each patient than most of our clinical colleagues and that time is comparatively cheap. An added bonus is that our dual skills in both education and exercise allow us to integrate challenging information with experiential learning as we supervise our patients’ guided experimentation with recovering movement and re-engaging with exercise.

## The pain sciences have clarified what physiotherapy treatments do and what they do not do

The ideal placement of physiotherapy to deliver the first-line treatments of education and exercise has substantially raised the profile and scientific credibility of the profession. In South Africa, physiotherapists have been among the first to seek out pain training in large numbers: physiotherapists are the most represented profession at the annual PainSA Congress and Pain Academy meetings and at professional development courses run by the Train Pain Academy. This may reflect a worldwide trend that physiotherapists are more likely to have updated pain knowledge than other healthcare professionals (Madden & Moseley 2016). It is our belief that undergraduate students studying physiotherapy in South Africa currently remain more likely to receive training in modern pain knowledge than their peers who are studying other professions. Physiotherapy arguably has a history in expert-driven practice, with certain expert physiotherapists’ extraordinary manual skills being lauded and sought out. Some examples of this are the Maitland or Mackenzie approaches to joint mobilisation (Hengeveld & Banks 2013) and the Bobath approach to neurodevelopmental rehabilitation (Valvano & Long 1991). Like its sibling professions that also originated through expert opinions about manual therapies, physiotherapy is moving forward, away from this focus on discrete techniques, towards a more robust, more democratically available set of skills and knowledge (Stander, Grimmer & Brink 2018). We are now a scientifically attentive profession. However, with this shift comes a degree of uncertainty: are we expected to discard our manual skills? Are we expected to never touch our patients? Could we have been doing harm for all these years? In the context of these concerns, physiotherapists who have engaged with mechanisms of pain have also developed a deeper understanding of the non-specific effects of treatments.

It is now widely acknowledged that every therapeutic interaction has a distinct, active treatment effect that is separate from the effect of the intervention itself. This ‘non-specific’ or ‘meaning’ response is a biopsychosocial phenomenon that can be helpful or harmful and may be the most potent ingredient in many inert treatments that were traditionally believed to have been active (Hutchinson & Moerman 2018). The meaning response is influenced by the quality of relationship between a patient and a clinician, as well as the broader context for the treatment (Benedetti 2013). Research on meaning responses (or placebo analgesia) has revealed that using good, ethically sound communication strategies to optimise the meaning response while also providing an active, evidence-based intervention could boost the clinical gain from physiotherapy treatments, to the benefit of the patient (Miller & Colloca 2009). The same understanding has also led to a robust debate about the efficacy of many physiotherapy treatments, including ‘trigger point’ therapy (Dommerholt & Gerwin 2015; Quintner, Bove & Cohen 2014) and dry needling (Boyles et al. 2015; Venere & Ridgeway 2016), and to a more parsimonious use of them: pain research has shed light on what certain manual treatments are doing and what they are not doing (Bialosky et al. 2011). Clinical trial design has also been influenced by awareness of the meaning response: physiotherapy treatments are now tested against viable sham treatments or established best practice, rather than against no treatment at all – precisely because a viable sham is expected to reproduce the non-specific meaning response to the clinical encounter without the active treatment.

## The pain sciences have expanded physiotherapy practice

While diligent application of Pain Science prunes the range of treatments that evidence-based physiotherapists use, the same process has added other treatment tools. The use of manual techniques is no longer indicated in the treatment of chronic widespread pain or in non-specific low back pain (Oliveira et al. 2018).

However, the accumulating evidence on the effects of mindfulness-based strategies (Hilton et al. 2016), ranging from diaphragmatic breathing to relaxation to mindfulness meditation, has motivated physiotherapists to return to these strategies as treatments. Whereas ‘breathing more deeply’ might have previously been recommended, the evidence supports the integration of mindfulness-based approaches for both acute and chronic pain as active and intentional physiotherapy treatments (Hilton et al. 2016).

Alongside this progress in clinical reasoning and treatment, a certain amount of ethical credibility has also been gained. Anecdotally, physiotherapists now seem to successfully discharge patients to self-management more often than before, presumably because patients are more empowered by their understanding of pain and feel less reliant on manual techniques that can only be obtained in the physiotherapist’s rooms. Several studies of physiotherapy treatments for chronic pain conditions using integrated exercise and educational approaches have demonstrated improvements in patients’ self-efficacy (Jönsson et al. 2018; Parker, Jelsma & Stein 2016; Saw et al. 2016). Improvements in self-efficacy are strongly associated with improved self-management with better use of healthcare services and reduced time off work (Lorig et al. 2001). This focus on empowering the patient to successfully self-manage is likely, over time, to beneficially alter the expectations that both the physiotherapist and the patient have of the treatment process.

Patients may increasingly expect to be educated, be empowered and have their independence fostered by a physiotherapist; physiotherapists may increasingly see it as their duty towards a patient to engender confidence in the robustness and the healing capabilities of the body. Similarly, the changes wrought by the uptake of Pain Science into clinical practice may cultivate interdisciplinary collaboration – particularly considering that interdisciplinary (rather than multidisciplinary) treatment is the best standard in clinical care for complex pain (Gatchel et al. 2014).

## The pain sciences have embedded interdisciplinary teams

Both multidisciplinary (MDT) and interdisciplinary teams (IDT) include the patient and various healthcare professionals. Pain management guidelines indicate that, at a minimum, these teams should include the patient, a physiotherapist and a physician (Gatchel et al. 2014). However, there are distinct differences in how MDT and IDT function and in the quality of care they deliver. When working in teams, the idea that all team members should ‘sing from the same song sheet’ is frequently put forward (Gatchel et al. 2014). This is critical to the successful treatment of pain, because conflicting information from healthcare professionals is a known iatrogenic contributor to pain. For example, if a person with an episode of acute low back pain is advised to rest by the doctor (which may be appropriate in an inflammatory phase), but is later advised to exercise by the physiotherapist (because their healing is now progressing) without clear clarification that the advice has changed because there has been time for healing, then confusion and distress may arise, fostering further pain and possible avoidance of activities due to fear of pain or further injury.

A multidisciplinary team can be likened to an orchestra, where all musicians are following the same song sheet, but each has a clearly delineated role, the conductor is never challenged and the patient is a passive listener. An interdisciplinary team can be likened to a jazz band, where the musicians follow the same song sheet but roles are flexible, while each has a preferred instrument (i.e. remains in their scope of practice). In the IDT, there is a blurring of roles and a fluidity in leadership with the leadership role passing from one to another, with active participation of the patient contributing to the form of the music. In this interdisciplinary jazz band model of healthcare, the physiotherapist will often take a leadership role but will also mentor patients to enable them to ultimately take the lead. The pain sciences have increased our awareness of how we work in teams and emphasised the value of paying attention to inter-professional communication in order to foster patient-centred care.

Thanks to their expertise, knowledge, communication skills and insight into the scope of adjacent professions, physiotherapists who are well-versed in the clinical pain sciences would do well to initiate and grow the establishment of interdisciplinary practice for the treatment of people with complex pain. Working in a well-functioning interdisciplinary team carries the double joy of being both interesting and supportive alongside the challenge of being ironically difficult to obtain remuneration. Here, too, as expectations of pain treatment shift with time, funders may come to recognise the immense value of high-quality, low-cost interdisciplinary care for pain over that of the high-cost, high-risk, low-return care that is currently more readily funded. Again, the physiotherapy profession is well positioned, alongside people who themselves have pain, to advocate for this enlightenment on the grounds of science, knowledge and ethics.

## Conclusion

The pain sciences have advanced physiotherapy on several levels, from basic research exploring mechanisms of pain to influencing assessments, clinical reasoning and treatments. Ultimately, they have shifted the profession to adopt mechanism- and evidence-based approaches which are patient-centred. We encourage readers who have encountered new concepts when reading this article to take up the challenge of learning more about pain. We also encourage physiotherapists to engage in a reflective process of evaluating their own clinical reasoning process – do you think about mechanisms, or only about the presenting symptoms? And finally, we challenge physiotherapists to continually engage in a critical process of reflecting on treatment techniques by considering the mechanisms of action of each treatment and by weighing the evidence that the treatment carries a risk of harm against the evidence that it confers benefit.
